# A Fresh Look at Islet Isolation from Rabbit Pancreases

**DOI:** 10.3390/ijms251910669

**Published:** 2024-10-03

**Authors:** Ekaterina Vasilchikova, Polina Ermakova, Alexandra Bogomolova, Alena Kashirina, Liya Lugovaya, Julia Tselousova, Nasip Naraliev, Denis Kuchin, Elena Zagaynova, Vladimir Zagainov, Alexandra Kashina

**Affiliations:** 1 Federal State Budgetary Institution of Higher Education, Privolzhsky Research Medical University of the Ministry of Health of Russia, Nizhny Novgorod 603082, Russia; bardina_p@pimunn.net (P.E.); bogomolova_a@pimunn.net (A.B.); bystrova93@gmail.com (A.K.); liya.lugovaya@inbox.ru (L.L.); tselousova.julia@yandex.ru (J.T.); nasip_95_kg@mail.ru (N.N.); pomc.kuchin@gmail.com (D.K.); ezagaynova@gmail.com (E.Z.); zagainov@gmail.com (V.Z.); meleshina_a@pimunn.net (A.K.); 2 Federal State Educational Institution of Higher Educational Institution “National Research Nizhny Novgorod State University Named after N.I. Lobachevsky”, Nizhny Novgorod 603105, Russia; 3 State Budgetary Healthcare Institution “Nizhny Novgorod Regional Clinical Oncology Dispensary”, Nizhny Novgorod 603126, Russia; 4 Nizhny Novgorod Regional Clinical Hospital Named after N.A. Semashko, Nizhny Novgorod 603005, Russia; 5 Federal Scientific and Clinical Center for Physico-Chemical Medicine Named after Academician Yu. M. Lopukhin, Moscow 119334, Russia

**Keywords:** islets of Langerhans, isolation, insulin, rabbit, FLIM

## Abstract

Islet transplantation represents a promising therapeutic approach for diabetes management, yet the isolation and evaluation of pancreatic islets remain challenging. This study focuses on the isolation of islets from rabbit pancreases, followed by a comprehensive assessment of their viability and functionality. We developed a novel method for isolating islet cells from the pancreas of adult rabbits. We successfully isolated viable islets, which were subsequently evaluated through a combination of viability assays, an insulin enzyme-linked immunosorbent assay (ELISA), and fluorescence lifetime imaging microscopy (FLIM). The viability assays indicated a high percentage of intact islets post-isolation, while the insulin ELISA demonstrated robust insulin secretion in response to glucose stimulation. FLIM provided insights into the metabolic state of the islets, revealing distinct fluorescence lifetime signatures correlating with functional viability. Our findings underscore the potential of rabbit islets as a model for studying islet biology and diabetes therapy, highlighting the efficacy of combining traditional assays with advanced imaging techniques for comprehensive functional assessments. This research contributes to the optimization of islet isolation protocols and enhances our understanding of islet functional activity dynamics in preclinical settings.

## 1. Introduction

Islet transplantation shows great promise for treating type 1 diabetes, with advancements such as the Edmonton protocol and FDA approval of donislecel indicating its growing acceptance [1,2,3]. As of 2022, an impressive 94 institutions and 5 networks have been identified as performing islet allotransplantation [4,5]. However, there are several challenges that hinder the widespread use of islet transplantation, such as a shortage of donor organs, ischemic conditions at the transplantation site, and immune reactions to foreign islets [6,7].

To address these problems, two main directions of research are being developed: in-creasing the number of islets [8,9,10] or finding new sources of insulin-producing cells [11,12,13,14], including the promising application of islet xenotransplantation [11,15,16,17,18,19], and improving transplantation efficiency through alternative sites [12], enhanced oxygen delivery to transplanted islets [20], the use of drugs to increase beta cell activity [21], or the use of decellularized matrices to support islets [12,22]. In parallel, studies on new immunological approaches [23] and the development of innovative encapsulation methods for immunoisolation [24] are being carried out.

Animal islets serve as models for research and alternative insulin sources, with the donors primarily being mice, rats, pigs, and primates [25,26,27,28]. While rats and mice are common in studies [29], primates closely model human diabetes [18,30], and pigs are used both in research and clinically, particularly in xenotransplantation techniques developed in Asian countries [11,15,17,31,32]. However, the use of rabbits is rare in modern studies of type 1 diabetes.

Rabbits have been used in laboratory research on type 1 diabetes and islet isolation, but to a much more limited extent compared to other animals. Research on islet isolation and transplantation in rabbits remains rare, despite their large size and ease of maintenance [33,34,35]. Major studies were conducted in the 1980s and focused on the isolation of islet cells [18,19,36,37]. Since then, the number of rabbits used in research in the U.S. has decreased from 550,000 to 230,000 per year, which is related to the rise in genetically modified rat and mouse models [35]. This has led to a decline in rabbit-specific studies, especially in the field of islet isolation [38].

Currently, rabbits are used as a reliable model for inducing diabetes [39]. Some studies have employed incomplete protocols for islet isolation from newborn rabbits based on methods for mice [40,41], while the use of adult rabbits as islet donors is limited. Nevertheless, macroencapsulated islets from newborn rabbits have been transplanted into patients with type 1 diabetes, and positive results were observed in 73.7% of the recipients after two years [42].

We believe that rabbits are an undervalued resource of islets. Our study aimed to address the existing knowledge gap regarding rabbit islets. We developed a novel protocol for isolating islets with an application to adult rabbit pancreases. Our protocol is based on modern rodent islet isolation approaches [29]. However, our procedure does not require the researcher to perform complex surgical manipulations associated with perfusion of the common bile duct, which significantly simplifies the entire procedure. Finally, we performed a comprehensive analysis of the isolated islets and assessed the morphological and functional characteristics of the obtained islets and their metabolic profile. The data we present will help advance diabetes research, including understanding the mechanisms of disease development, improving the quality of islets after isolation, and possibly opening up a new source of cells for xenotransplantation.

## 2. Results

### 2.1. Sequence Analysis of Insulin and C-Peptide

Amino acid sequence analyses of insulin and C-peptide are crucial for several reasons; for example, the specific sequences determine the biological activity and function of insulin and C-peptide, influencing how they regulate glucose metabolism and insulin signaling. Knowledge of the insulin structure is essential for producing recombinant insulin for diabetes treatment and for designing insulin analogs with improved properties [43]. Also, an amino acid sequence analysis plays a critical role in ensuring the success and safety of islet xenotransplantation by addressing the compatibility, immune response, and functional efficacy [44,45].

When comparing the insulin genes of rabbits to those of other animals, notable differences and similarities emerge. In rats (P01322; P01323), insulin genes form a two-gene system that is significantly distinct from the rabbit and human versions (Table 1). To analyze the similarity of insulin and C-peptide among rabbits, humans, pigs, and rats, we examined the putative amino acid sequences.

According to the Basic Protein Local Alignment Search Tool (BLAST) and UniProt, the amino acid sequence of the a-chain of insulin in rabbits (P01311) is identical to those of pigs (P01315) and humans (P01308) (Table 1). The b-chains of insulin in these species are nearly identical as well, differing by only a single amino acid. In contrast to insulin, the C-peptide sequence of all three animals differs significantly, but only the pig C-peptide has a deletion of two amino acids. At the same time, the rabbit C-peptide sequence has a similar number of amino acids to the human C-peptide sequence, although, like the porcine C-peptide, it has differences in the amino acids themselves. Thus, despite the differences, the rabbit C-peptide is closer to the human one than the porcine one.

### 2.2. Morphological Features of Rabbits

**Figure 1:**
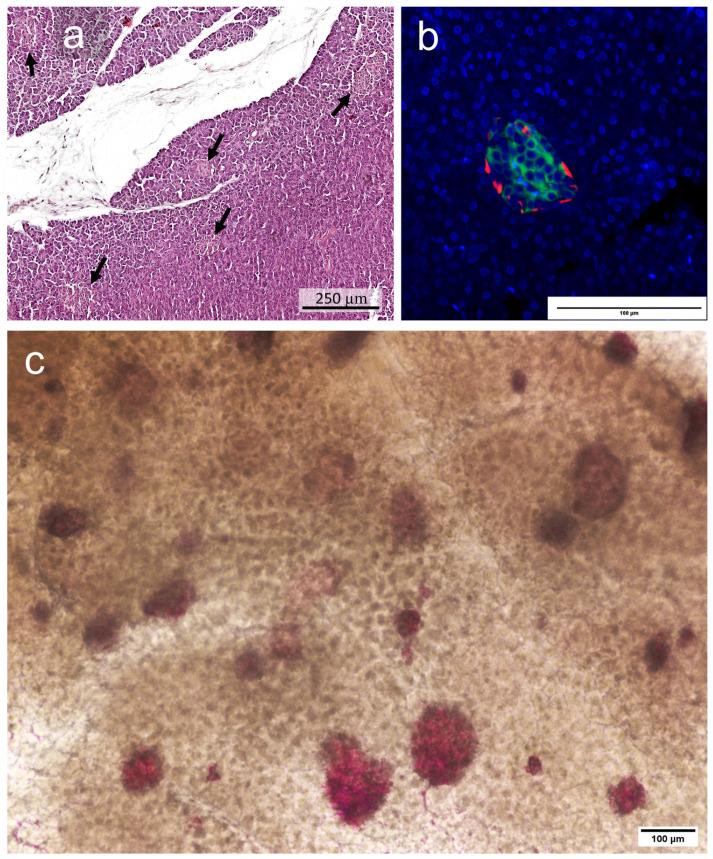
An example of islets in the pancreatic tissue. (**a**) Hematoxylin and eosin staining; black arrows point to the islets. (**b**) IHC staining; insulin (green), glucagon (red), DAPI (blue). (**c**) Dithizone staining.

The overall yield from the pancreas of rabbits can vary greatly depending on the breed and specialist chosen. Our research was carried out on rabbits of the Gray Giant breed. After a histological analysis of pancreatic sections, the total number of islets was determined. The rabbit pancreas contains about 25 × 10^4^ islets [46]. Figure 1a,c show the distribution of islets per pancreatic tissue, with a ranged size. In this case, Figure 1a demonstrates the staining of islets on a histological section of the pancreas, while Figure 1c demonstrates the staining of islets using specific staining with dithizone. The immunohistochemical analysis showed that the islets in the tissue are similar in structure to the islets of rodents: beta cells fill the core, while alpha cells are located at the periphery (Figure 1b).

**Figure 2:**
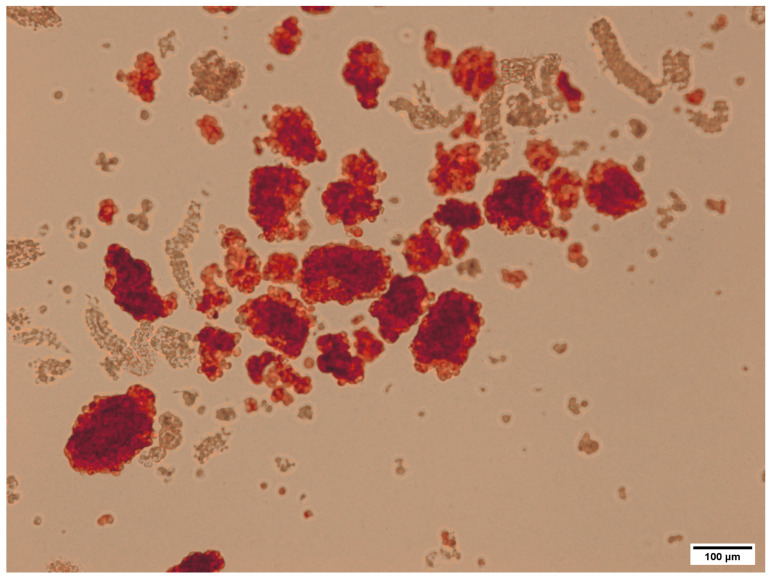
An example of islets immediately post-isolation and purification; dithizone staining.

The average islet yield from the pancreas of one 8–10-week-old rabbit was 4836 ± 604, with a purity ranging from 70% to 95% (Figure 2). This result is consistent with the literature data, since the islet yield is 10 to 30% of a typical pancreas [29]. However, the pancreas of rabbits contains almost 10 times more islets. This high concentration of islets suggests that fewer animals are needed for research purposes, making it a more efficient model for studying pancreatic function and related diseases. Additionally, the structural similarity of the rabbit islets to those of rodents reinforces their potential as a suitable alternative in biomedical research.

**Figure 3:**
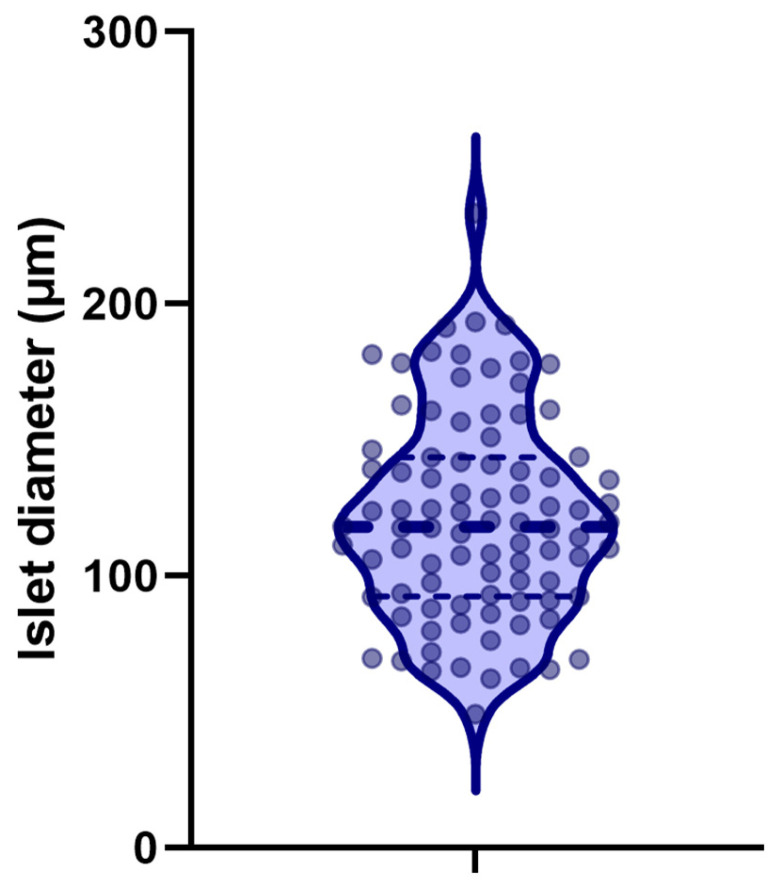
A violin plot depicts distribution of rabbit islet size after isolation. The dots represent each islet’s diameter measurement. The solid line indicates the diameter, while the dotted lines indicate the quartiles.

The islet size was determined using a Leica microscope (Deer Park, IL, USA) after isolation by dithizone staining. The mean diameter of the rabbit islets obtained was 121.4 μm (SD ± 37.4). The range for the islets was 50–250 μm. As Figure 3 shows, the size of the isolated islets ranged from 50 to 250 μm and the shape was irregular.

### 2.3. Functional Features of Rabbits

#### 2.3.1. Islet Viability

**Figure 4:**
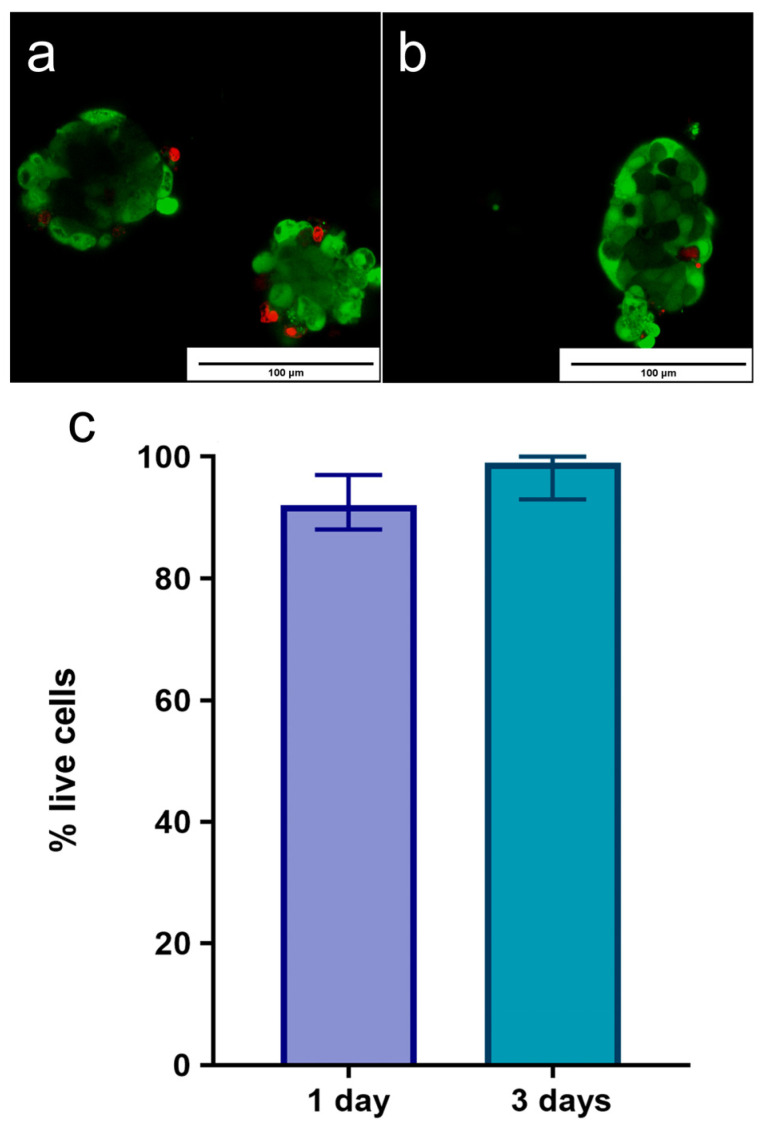
An example of viability as measured by calcein-AM and propidium iodide fluorescence on the first day after isolation (**a**) and on the third day after isolation (**b**). Calcein-AM is depicted with green fluorescence and propidium iodide is depicted with red fluorescence. (**c**) Assessment of the viability of rabbit islets on the 1st and 3rd days after isolation. The increase in viability was caused by a decrease in the number of cells, in particular, dead cells, during incubation in the culture medium.

After 24 h of culture, the percentage of viable cells within each islet was measured by live/dead fluorophores, using confocal microscopy. Figure 4a,b show an image captured with the confocal microscope, showing islets stained for viability. The live/dead stain shows live cells (green) with single dead cells (red) in the islet. As seen in the representative live/dead images (Figure 4c), the isolated islets retained their viability. A quantitative image analysis of the live/dead results measured a high percentage of viable cells (91 (78; 97)) at 24 h. A similar trend in the percentage of islet viability was observed at 72 h (99 (92; 100)), although significant differences were not measured between the two time points. Notably, an increase in the islet viability percentage was measured between 24 and 72 h, likely due to the natural clearance of dead cells from the culture over time.

#### 2.3.2. Insulin Secretion and Glucose Stimulation

**Figure 5:**
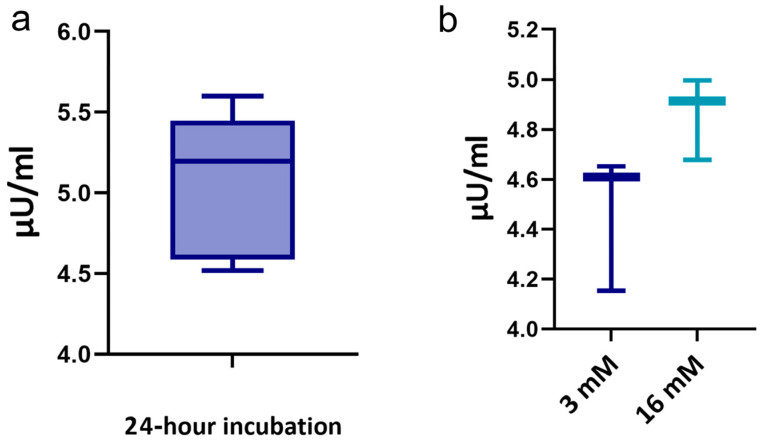
Evaluation of the functional activity of rabbit islets. (**a**) Daily secretion of insulin by rabbit islets on the day after isolation. (**b**) Functional assessment of islets by glucose-stimulated insulin response for differences between insulin secretion during low versus high glucose challenge.

A simple method for comparing the insulin-secretion-per-islet equivalency is to evaluate the ratio of islet insulin release. Static insulin secretion experiments were completed with equal rabbit islets by using an insulin-sensing ELISA. During one day of incubation in the culture medium, 350 islets produced 5.19 [4.53; 5.59] μU/mL of insulin. The ELISA showed that the islets secreted 4.6 [4.16; 4.64] μU/mL insulin at a glucose concentration of 3 mmol/L. The islets responded appropriately to the incubation conditions (Figure 5). Stimulation with high glucose resulted in a release of insulin that was 1.08 times higher than the basal levels (4.91 [4.69; 4.97] μU/mL). The insulin levels returned to basal amounts with a return to the low-glucose environment.

Overall, these findings suggest that the pancreatic islets from Gray Giant rabbits are not only viable, but also functionally active, making them a promising model for studying insulin secretion and related metabolic processes in research settings. The ability to maintain a high viability and functional responsiveness under culture conditions underscores their potential utility in diabetes research and therapeutic applications.

### 2.4. FLIM (Fluorescence Lifetime Imaging)

Pancreatic islet cells are cells with high metabolic activity, which is associated with their narrow specialty, namely the secretion of insulin in response to stimulation by glucose. These complex biochemical processes occurring in cells are directly reflected in their energy metabolism, which can act as an indicator of the viability and functional state of cells.

Over the past decade, FLIM has proven itself to be a highly sensitive non-invasive method for analyzing the energy metabolism of cells by recording the fluorescence lifetimes of the metabolic cofactors reduced NAD(P)H and oxidized FAD [47,48,49,50,51]. The advantages of this approach are the absence of the need for sample staining, the ability to directly visualize the metabolic status, and the high molecular specificity of the method. All of this makes it possible to study living cells and tissues with minimal sample preparation and exposure to cells. It is important to note that the fluorescence lifetime depends on the molecular interaction of the fluorophore, not on its concentration. FLIM NAD(P)H allows us to distinguish between free and protein-bound forms of the cofactor [49]. The short (τ1, α1) and long (τ2, α2) components of the NAD(P)H fluorescence lifetime decay are associated with the free and protein-bound states of the cofactor, respectively. An increase in the proportion of free NAD(P)H in the cytosol is associated with glycolytic metabolism, while a high ratio of bound to total NAD(P)H is largely associated with mitochondria and suggests a metabolic state characterized by increased oxidative phosphorylation. Previously, using FLIM, it was shown that diabetic islet cells have a more glycolytic metabolism compared to healthy ones [52]; glucose stimulation has different effects on healthy β- and α-cells and cells with type 2 diabetes [53].

In this study, we analyzed the contribution of the main metabolic pathways (glycolysis and OXPHOS) to the total energy metabolism of islet cells using the fluorescence lifetime analysis (τm, τ1, τ2) and the fluorescence lifetime contributions (α2 and α1/α2) of the free and bound forms of NAD(P)H using FLIM.

**Figure 6:**
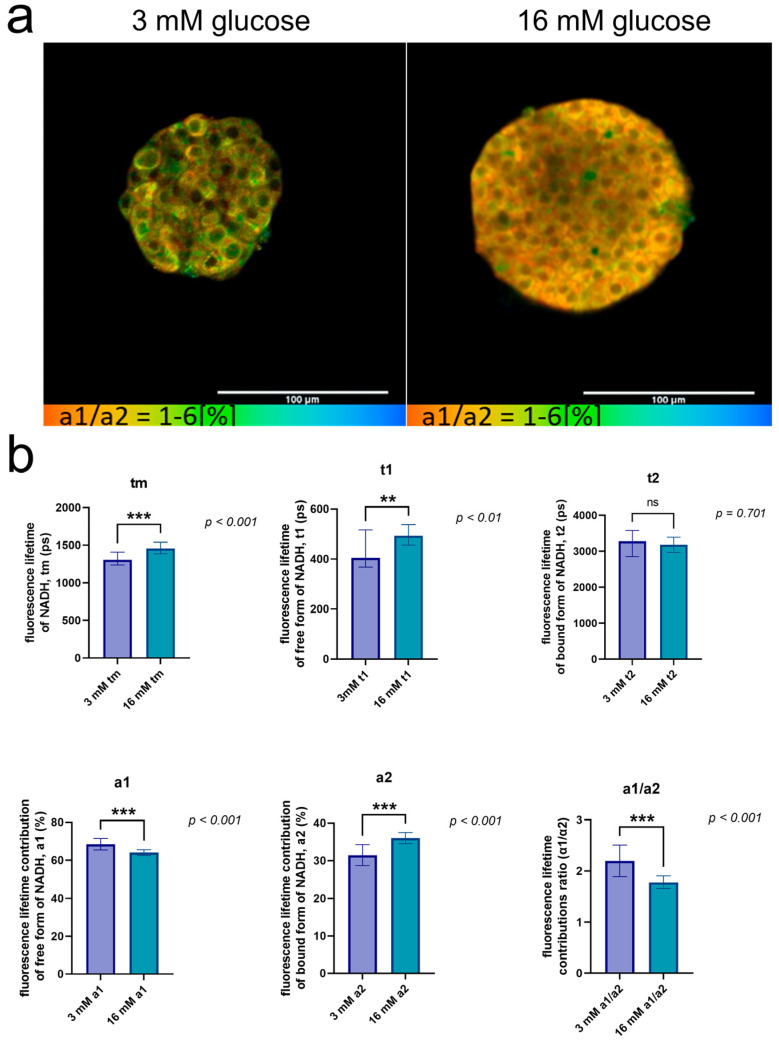
FLIM of NAD(P)H in isolated islets of rabbit. (**a**) Pseudo-color-coded images of α1/α2 of the NAD(P)H in pancreatic islets of rabbit by glucose-stimulated insulin response (scale bars: 1–6 (α1/α2)). The scale length in all pictures is 100 µm. (**b**) Mean values of τm, τ1, τ2, α1, α2 and α1/α2 of the NAD(P)H in pancreatic islets of rabbit by glucose-stimulated insulin response (mean ± SD). ***—*p* < 0.001 (α1, α2, α1/α2, τm), **—*p* < 0.01 (τ1).

An analysis of the FLIM data showed that the islet cells from rabbits in a high-glucose buffer differed from those in a low-glucose buffer for all the tested parameters (*p* ≤ 0.05) (Figure 6a). Namely, the islet cells from rabbits in the high-glucose buffer were characterized by lower values of τ2 and a1 and higher values of τm compared to the islet cells in the low-glucose buffer (Figure 6b). Several parameters were distinguished, with the differences between them being particularly pronounced. These were the parameters τm and τ1. Moreover, based on the values of α2 and α1/α2, we can assume that OXPHOS predominated in the islet cells from rabbits after incubation in the high-glucose buffer.

Using FLIM, we showed that rabbit islet cells exhibit levels of energy metabolism typical for healthy cells, which is in good agreement with other studies [53,54]. Thus, in further studies, FLIM can be used to determine the number of viable and metabolically active islets, as well as in the study of insulin-deficient states in pancreatic islet cells.

## 3. Discussion

The use of rabbits to isolate the islets of Langerhans has several important aspects, especially from the point of view of cell and molecular biology and medical research. Isolating the islets of Langerhans from rabbits allows us to study the molecular pathways involved in the secretion of insulin and other hormones. This is important for understanding how different molecules, such as glucose, lipids, and hormones, affect islet function. Rabbits have genetic and physiological characteristics that make them a suitable model for studying mammals. Rabbits offer a unique advantage as an intermediate-size model, positioned between rodents and larger, more costly animals such as pigs. Their size facilitates convenient blood sampling and provides access to a variety of cells and tissues from a single specimen [55]. Also, physiologically, rabbits on a high-fat diet display hemodynamic and neurohumoral changes akin to those observed in obese humans, along with a comparable lipid metabolism [56]. From a phylogenetic perspective, rabbits are more closely related to primates than to rodents [57]. Furthermore, rabbits tend to have longer lifespans than rodents, and their immune system genes show a greater similarity to human immune system genes compared to those of rodents [57,58]. Additionally, the insulin gene in rabbits is more similar to the human version than to rodent insulin genes (Table 1). Finally, the low success rates of translating certain mouse studies to human conditions indicate that intermediate animal models such as rabbits may often be more appropriate [55]. This allows researchers to conduct experiments that may be more relevant to humans compared to other models, such as mice or rats. One of the significant advantages of using rabbits as a source of islets is the relatively small size of their pancreas compared to other animals, which results in a higher concentration of islets. This allows for the production of a larger number of islets using a minimum amount of reagents, making the process more efficient and cost-effective. Additionally, rabbits are affordable animals with a rapid growth rate, allowing researchers to produce significant numbers of islets in a relatively short period of time.

The rabbit pancreas is an attractive source of islets. As far as we know, until the end of the 20th century, rabbits have been widely used as experimental animals in diabetes research, including as a source of islet cells [59]. In addition, there is a study in which rabbit islets were successfully used as a source of insulin in the xenotransplantation of microcapsules into patients with type 1 diabetes. Positive results were observed in 73.7% of the recipients during the 2-year follow-up period [42]. However, more recently, rats and mice have gained the greatest popularity due to the appearance of a large number of genetically modified animals, as well as pigs, due to their size and the similarity of the structure of the digestive system to the human. Over the past 30 years, using islets derived from these animals, numerous discoveries have been made in the field of transplantation technologies for the treatment of type 1 diabetes mellitus and pancreatogenic diabetes, including the development of xenotransplantation [12,22,23,24].

The production of human-like insulin has become one of the main reasons for the active use of pigs as the primary source of islets for research and xenotransplantation. Research strongly suggests that porcine-derived islets may be a promising replacement for human islets in the treatment of T1DM [16]. It is worth noting that the amino acid sequences of rabbit and pig proinsulin are quite close to human ones. According to the Basic Protein Local Alignment Search Tool 2.13.0 (BLAST) and UniProt, rabbit insulin (P01311), like porcine insulin (P01315), differs from human insulin (P01308) by only one amino acid at position 30 of the B chain. Porcine insulin is characterized by the presence of non-polar alanine. On the other hand, rabbit insulin contains serine, which is a polar, uncharged amino acid similar to threonine in human insulin. It is known that threonine and serine are closely related amino acids with a similar reactivity [60]. Thus, the differences between rabbit and human insulin are less significant compared to porcine insulin.

In addition, there have been recent reports highlighting the key role of C-peptide in the pathogenesis of type 1 diabetes. Clinical studies have shown that the administration of C-peptide to patients with type 1 diabetes who lack this peptide results in the improvement of diabetes-related renal and nerve dysfunction [61,62]. Porcine C-peptide is significantly different from human C-peptide, including a deletion of two amino acids (Table 1). This difference may result in an immune response during xenotransplantation and potentially reduce the effectiveness of porcine islet treatment. Rabbit C-peptide has fewer differences from the human protein, and it also consists of 110 amino acids. Therefore, when transplanting rabbit islets, one can count on a high efficiency of treatment using xenotransplantation.

Overall, the use of rabbits as islet donors in experimental and clinical settings offers several advantages over rats and pigs. Compared to rats, the rabbit pancreas is about the same size, but is denser in islets, so the islet yield is increased when using the same amount of expensive enzyme. The delicate nature of porcine islets poses challenges during the isolation process, as they tend to disintegrate into individual cells [63,64]. On the contrary, the stability and easy isolation of rabbit islets provide a significant advantage in the use of these animals.

We developed a rabbit islet isolation protocol based on the current protocols for isolation from small laboratory animal pancreases [29], adapting it for adult rabbits. Our isolation protocol relies on the mechanical fragmentation of the pancreas instead of perfusion, allowing for the efficient delivery of collagenase to the pancreatic tissue. Re-fermentation after tissue cooling ensures the preservation of the maximum number of intact islets. The minimal damage to islets and high yield achieved with a reduced effort set our method apart, making rabbits an appealing choice for experimental studies. Ultimately, we provide a comprehensive technique for extracting islets of Langerhans from rabbit pancreases. Our results demonstrated a high yield of islets from a single pancreas. Compared to existing protocols (about 600 islets of Langerhans per rat [29] and 1786 ± 350 islets per gram of pig pancreas [15]), and considering that the weight of a rabbit pancreas is approximately 1 g, our isolation results are quite attractive. Our rabbit islet isolation protocol with well-controlled digestion times and carefully performed purification steps yielded an average of 4836 ± 604 healthy islets. Despite the similar structure of insulin and C-peptide, as well as the high yield of islets per pancreas, there are limitations associated with obtaining sufficient rabbit islets for human xenotransplantation. On average, obtaining sufficient islets for human therapy would require a significant number of rabbits, based on data from pig islet xenotransplantation [65]. In his study, Prochorov used nine rabbits for one human transplant [42]. However, it is worth noting that the easier care of rabbits and less labor-intensive islet isolation may contribute to the increased interest in using rabbits to obtain islets for research purposes. The high concentration of islets in the pancreas indicates that fewer animals would be required for research, making it a more efficient model for studying pancreatic function and related diseases. For experiments such as those with induced diabetes, various metabolic parameters, etc., a large number n per experimental group, may be required, which, together with the low cost of maintaining rodents, may be a limiting factor for the use of rabbits in these experiments. However, in vitro studies of the islets themselves, as well as xenotransplantation models from rabbits to rodents, will provide a significant reduction in the number of animals used in an experiment.

Rabbit islets have a peri-islet membrane, which ensures their integrity and viability after isolation. The viability of isolated islets tends to be 100% by day 3 of observation. The current islet isolation protocols in rats and mice demonstrate a 90–95% failure of isolated islets [29,66]. The insulin secretion amount after overnight incubation in a complete RPMI-1640 medium containing 5.5 mM glucose was 5.19 [4.53; 5.59] μU/mL. Similarly, the insulin secretion from non-transfected control islets may be within these limits (5.89 ± 1.61) [67]. The GSIS results may vary when using different protocols. We used a single-islet stimulation method with 16 mM glucose, analyzing 15 islets on the day after isolation. The glucose stimulation index (1.08) may be low compared to some literature data on rodent islets. The limited use of rabbits is the reason for the lack of information on the GSIS analysis. However, our results are similar to the limited data in the literature [38]. Also, the SI of the islets isolated by us was above one, which, according to Glieberman et al. and Di Piazza et al., is sufficient to prove the functional activity of the islets [68,69]. Furthermore, Ricordi et al. reported that some lots of islets actually failed the outlined GSIS criterion (stimulation index < 1), even though these lots had been used for transplantation in patients [70]. According to studies, insulin secretion by the islets may differ depending on the chosen assessment method [71]. Thus, we confirmed insulin secretion not only by an ELISA. The data obtained, together with dithizone staining, demonstrated the preservation of insulin secretion by the isolated islets. Thus, this study most fully describes not only the protocol for obtaining, but also an assessment of the quality of the isolated rabbit islets, allowing for a full comparison with the results of islet isolation from other organisms.

It has previously been shown that, in healthy β-cells, an increase in the glucose concentration contributes to a proportional increase in the ATP levels in the cells as a result of OXPHOS, while in β-cells with pathology, increased glycolysis is observed [53,54]. It has been previously shown that high bound/total NAD(P)H indicates a metabolic state with high oxidative phosphorylation, whereas low bound/total NAD(P)H indicates predominant glycolysis. The metabolic trajectory was as expected, with the bound/total NAD(P)H in INS-1E cells moving from a relatively low value at low glucose concentrations to an elevated value upon glucose stimulation. Thus, such features of cell energy metabolism can be used as indicators for diagnostic purposes. The use of metabolic imaging technologies based on FLIM and the autofluorescence of the intracellular metabolites of NAD(P)H opens up wide possibilities for minimally invasive and non-contrast diagnostics of pancreatic islet quality and for determining the number of viable and metabolically active islets. The FLIM analysis revealed differences in all the parameters when the islets were stimulated with glucose. Changes in the fluorescence lifetime contributions showed that the contribution of OXPHOS increased after stimulation by 16 mM glucose. Interestingly, the metabolic shift towards oxidative phosphorylation after glucose stimulation observed in β-like cells was recently confirmed in the work by Wang and collaborators [53]. Thus, the islets, after isolation according to the protocol we developed, react in accordance with healthy islets [52].

Thus, the use of rabbits for the isolation of islets of Langerhans provides unique opportunities for research in the field of molecular biology and diabetology. This allows for a deeper understanding of the pathogenesis of diseases, the development of new treatment methods, and an improvement in the quality of life of patients with carbohydrate metabolism disorders. By developing a robust rabbit islet isolation protocol, we want to unlock the potential of rabbits as a valuable source of islets for research and advanced islet transplantation technology as a treatment option for people with type 1 diabetes.

## 4. Materials and Methods

### 4.1. Animals

A series of experiments was carried out on male Gray Giant rabbits weighing 2–3 kg (n = 23). Eight-week-old rabbits were housed under standardized light–dark cycles in a temperature-controlled, air-conditioned environment under specific pathogen-free conditions with free access to food and water. All the experiments were approved by the local ethics committee of PRMU (protocol No. 10, dated 26 June 2020).

Before the procedure, the rabbits were anesthetized with Zoletil, 5 mg/kg (Virbac, Hamilton, New Zealand), and xylazine, 4 mg/kg (Bayer, Leverkusen, Germany). Various methods of anesthesia were used to ensure that the research protocols were consistent with humane methods. In our laboratory, we used zoletil/xylazine for intraperitoneal injections.

### 4.2. Islet Isolation

To begin the pancreas isolation protocol, prepare 25 mL of tissue preservation solution (Hank’s solution (Sigma-Aldrich, product No. H6648, St. Louis, MO, USA) with 5% BSA (Sigma-Aldrich, 9048-46-8) and 1% antibiotic–antimycotic (ThermoFisher, 15240062, Waltham, MA, USA)) in a 50 mL conical tube on ice, along with additional tubes of wash solution (Hank’s solution (Sigma-Aldrich, product No. H6648, St. Louis, MO, USA) with 0.5% BSA (Sigma-Aldrich, 9048-46-8, St. Louis, MO, USA) and 1% antibiotic–antimycotic (ThermoFisher, 15240062, Waltham, MA, USA)) to inhibit self-proteases. Avoid using cold accumulators that are colder than ice to prevent tissue damage. Weigh the rabbit and administer anesthesia intramuscularly at 100 µL per kg. Once the rabbit is fully anesthetized, remove the fur from the abdomen and euthanize using guillotine decapitation. Disinfect the area with 70% alcohol, and then make a midline incision to access the abdominal cavity. Carefully isolate the pancreas, lifting the stomach to free it from surrounding tissue, and extract it into the preservation solution. Limit the number of rabbits to ten to keep warm ischemia under 5–7 min.

After collection, transfer the tube to a sterile room, remove visible fat with scissors, and cut the pancreas into 1–2 mm fragments. Wash the slices by centrifuging for 2 min at 300× *g*, discarding the supernatant and resuspending the sediment in Hank’s solution. Repeat this washing step 2–4 times until blood contamination is cleared, keeping all manipulations on ice to preserve viability.

To initiate pancreatic fermentation, prepare an enzymatic digestion mixture by combining 10 mL of warm Hank’s solution (Sigma-Aldrich, product No. H6648, St. Louis, MO, USA) (37 °C) and 1 mL of collagenase solution (0.1 g of collagenase V (Sigma-Aldrich, cat. No. 9001-12-1, St. Louis, MO, USA) in 10 mL of Hank’s solution) per 10 mL of pancreatic tissue sediment. The mixture should be incubated for approximately 11 min at 37 °C with slight agitation at 150 g.

After incubation, take a 200 μL sample of the solution and place it on a plate, adding 1–2 drops of dithizone solution (10 mg of dithizone (Sigma-Aldrich, cat. No. 60-10-6, St. Louis, MO, USA) in 1 mL of DMSO (Sigma-Aldrich, cat. No. 67-68-5, St. Louis, MO, USA)). This staining process allows for the identification of pancreatic islets, which will appear crimson under a microscope. At this stage, you should observe loose tissue along with a small number of free islets. This visual assessment is essential to determine the effectiveness of the enzymatic digestion prior to diluting the collagenase.

If more than 50% of the islets remain attached to acinar tissue, add an additional 0.5 mL of collagenase solution per 10 mL of tissue and incubate for an additional 2–3 min at room temperature before repeating the washing process. After completing the collagenase wash, allow a break of 30–60 min while keeping the test tube with the sample at 4 °C. This resting period helps to achieve an aseptic solution of the digested pancreas and ensures even cooling of the tissue, which enhances the quality of subsequent cleaning. After this break, transfer the test tube to a clean workspace for further processing.

To halt the enzymatic digestion, quickly dilute the solution by adding wash solution. Centrifuge the digested tissue at 300× *g* for 2 min, and then carefully collect the supernatant and resuspend the sediment in wash solution. Repeat this washing step three times, ensuring that all manipulations are conducted using cold solutions and that centrifugation occurs at 4 °C to maintain tissue viability. Finally, reassess the digested tissue by staining it again with dithizone.

The next step is to filter the digested pancreatic tissue through a cell screen into 50 mL conical tubes. Resuspend the remaining tissue in wash solution and filter again until only a small clot remains (ideally <2 mL). Centrifuge for 2 min at 300× *g*, removing the supernatant.

Resuspend the pellet in a 1:4 solution of Ficoll 25% (dissolved Ficoll 400 (Sigma-Aldrich, cat. No. 26873-85-8, St. Louis, MO, USA) in Hank’s solution) using a 10 mL pipette. Sequentially layer 5 mL each of Ficoll 20%, 15%, and 11% solutions, and then centrifuge for 15 min at 800× *g*. Islets should appear as floating white spots between the layers. If fewer islets are observed, check the sediment for any remaining islets.

Transfer the supernatant to new tubes with wash solution and centrifuge at 300× *g* for 4 min. Discard the supernatant, and then wash the pellet with wash solution. Decant and replace with prewarmed RPMI1640 medium (ThermoFisher, 11875093, Waltham, MA, USA) (with 10% FBS and 1% Pen/Strep, maintaining glucose at 1 g/L).

Finally, transfer the cleaned islets to sterile culture mats at a density of no more than 300 islets/mL and place in a recovery incubator for optimal growth. This method ensures the proper isolation and preparation of islets for further use.

### 4.3. Assessment of the Belonging of the Derived Cells to the Islets of Langerhans, the Number of Isolated Islets, and Their Purity and Size

To assess the presence of islets of Langerhans in the isolated cells, the number of isolated cells and their purity and size were determined using dithizone staining [72]. A 100 μL sample was taken from the isolated islet suspension. An amount of 1–2 drops of dithizone solution was added to the sample. To accurately determine the number of isolated islets, the volume of the original islets was used. The purity of the isolated islets was assessed by calculating the percentage of cells that showed positive dithizone staining. The islet size was assessed using the ImageJ software. The calculation of the islet equivalent value (IEQ) was carried out according to the classical method [73].

### 4.4. Immunohistochemistry

Pieces of the pancreas were fixed in formalin and subsequently embedded in paraffin. Four-micrometer slices of the pancreas are susceptible to immunofluorescence staining. Immunofluorescence double staining was performed using a primary antibody of glucagon monoclonal (1:10; Invitrogen, Carlsbad, CA, USA) and insulin monoclonal antibody (1:100; Invitrogen, Carlsbad, CA, USA) overnight at 4 °C. The samples were counterstained with DAPI (1:1000; BioLegend, San Diego, CA, USA) according to the manufacturer’s protocol. The percentage of beta cells was calculated using the ImageJ 1.43 u program (NIH, Bethesda, MD, USA), as the ratio of the area of beta cells to the area of the entire islets.

### 4.5. Viability Assessment

The cell survival and death of the isolated islet cells were assessed by the Live/Dead Cell Double Staining Kit (Sigma-Aldrich, 04511-1KT-F, St. Louis, MO, USA) at 24 and 72 h after isolation according to the standard protocol. The 500 μL suspension of islets was incubated in a mixture of 2 μL calcein-AM (live cell, green) and 1 μL ethidium homodimer-1 (dead cell, red) for 15 min at room temperature. Visualization was performed on an LSM 880 confocal fluorescence microscope (Carl Zeiss, Oberkochen, Germany). For calcium-AM detection, the fluorescence was excited to detect 488 nm waves, with detection in the range of 500–549 nm, and the propidium iodide fluorescence was excited to detect waves of 543 nm, with detection in the range of 611–700 nm. The ratio of the green-colored area to the total cell area (%) was calculated from the confocal images of the aggregates in 3–5 randomly selected fields per sample using the ImageJ 1.43 u software.

### 4.6. Assessment of Functional Activity (Glucose-Stimulated Insulin Secretion (GSIS))

Samples of 15 islets were incubated at 37 °C and 5% CO_2_ for 1 h in each solution in the corresponding order: KRBH solution without glucose (0 mmol/L), low glucose (3 mmol/L), and high glucose (16 mmol/L). The supernatant was collected and stored at −20 °C for the analysis. The insulin concentration released during incubation was measured using an ELISA kit for rabbit insulin (Cloud-clonecorp, Katy, TX, USA, CEA448Rb). The absorbance was measured using a microplate reader with a 450 nm wavelength filter (BioTek Synergy Mx Microplate Reader SMA, Santa Clara, CA, USA) and expressed as μg/mL. The stimulation index (SI) was calculated as the ratio of the insulin concentration secreted in high glucose over the insulin concentration secreted in the low-glucose incubation.

### 4.7. Assessment of Metabolic Activity (FLIM Imaging Strategy)

The fluorescence and time-resolved images were captured using an LSM 880 (Carl Zeiss) microscope equipped with a state-of-the-art short-pulse femtosecond Ti:Sa laser called Mai Tai HP (Spectra-Physics, Milpitas, CA, USA). This laser had a pulse repetition rate of 80 MHz and emitted pulses lasting 140 ± 20 femtoseconds. Additionally, a FLIM system for time-resolved microscopy provided by Becker & Hickle GmbH (Berlin, Germany) was used. To capture fluorescence images of NAD(P)H, a two-photon excitation technique was employed at a wavelength of 750 nm. The resulting fluorescence was detected within the range of 455–500 nm. For FLIM imaging, the following parameters were utilized: an image size of 512 × 512 pixels and a field of view size of 213 × 213 μm^2^. To ensure accurate results, a minimum of 5000 photons per image pixel was required. All the experiments were conducted under the constant conditions of 37 °C and 5% CO_2_. To analyze the FLIM images, the SPCImage 8.3 software provided by Becker & Hickl GmbH was utilized. This software allowed us to determine the fluorescence lifetimes (τ1 and τ2 in picoseconds) and the contributions of each lifetime component (α1 and α2 in percentages).

### 4.8. Software

The software used included ImageJ 1.43u and the SPCImage 8.3 software, provided by Becker & Hickl GmbH.

### 4.9. Sourcing Sequences

The amino acid sequence of the insulin preproprotein from various sources (human, pig, and rabbit) was analyzed using the Basic Protein Local Alignment Search Tool (BLAST) and UniProt.

### 4.10. Statistical Analyses

The statistical analyses were performed using the Prism 9.4.0 software (GraphPad). Each experiment was repeated at least five times. The data are presented as the median, and the error bars represent the confidence interval. Two-tailed Mann–Whitney and unpaired *t*-tests were used to determine the statistical significance between groups; *p*-value ≤ 0.05.

## 5. Conclusions

Despite the existing protocols for islets from the pancreas of small laboratory animals and humans, the problem of obtaining a stable, high number of islets for both research and transplantation has not yet been solved. Current protocols focus primarily on the use of rodent and porcine pancreases. We present a protocol for quickly and efficiently obtaining large numbers of islets using rabbits. Using this protocol, it is possible to obtain a higher yield of isolated islets compared to the current state-of-the-art results. In addition, isolated rabbit islets retain a high viability and metabolic activity compared to the islets from other animals. Our research underscores the promise of rabbit islets as a valuable model for investigating islet biology and diabetes treatment. The data we provide will contribute to the advancement of diabetes research by enhancing our understanding of disease development mechanisms, improving the quality of isolated islets, and potentially offering a new source of cells for xenotransplantation.

## Figures and Tables

**Table 1:** Amino acid sequences of insulin from humans, rabbits, pigs, and rats.

**α-chain**	
*Homo sapiens* (P01308)	GIVEQCCTSICSLYQLENYCN
*Oryctolagus cuniculus* (P01311)	GIVEQCCTSICSLYQLENYCN
*Sus scrofa* (P01315)	GIVEQCCTSICSLYQLENYCN
*Rattus norvegicus* (P01322) *Rattus norvegicus* (P01323)	GIVDQCCTSICSLYQLENYCN GIVDQCCTSICSLYQLENYCN
**β-chain**	
*Homo sapiens* (P01308)	FVNQHLCGSHLVEALYLVCGERGFFYTPKT
*Oryctolagus cuniculus* (P01311)	FVNQHLCGSHLVEALYLVCGERGFFYTPKS
*Sus scrofa* (P01315)	FVNQHLCGSHLVEALYLVCGERGFFYTPKA
*Rattus norvegicus* (P01322) *Rattus norvegicus* (P01323)	FVKQHLCGPHLVEALYLVCGERGFFYTPKS FVKQHLCGSHLVEALYLVCGERGFFYTPMS
**C-peptide**	
*Homo sapiens* (P01308)	EAEDLQVGQVELGGGPGAGSLQPLALEGSLQ
*Oryctolagus cuniculus* (P01311)	EVEELQVGQAELGGGPDAGGLQPSALELALQ
*Sus scrofa* (P01315)	EAENPQAGAVELGGG__LGGLQALALEGPPQ
*Rattus norvegicus* (P01322) *Rattus norvegicus* (P01323)	EVEDPQVPQLELGGGPEAGDLQTLALEVARQ EVEDPQVAQLELGGGPGAGDLQTLALEVARQ

## Data Availability

The datasets used and/or analyzed during the current study are available from the corresponding author upon reasonable request. The amino acid sequences were performed by BLAST and UniProt (rabbit insulin (P01311), rat insulin (P01322), porcine insulin (P01315), and human insulin (P01308)).
